# Supplementary material for: Predictable evolution towards larger brains in birds colonizing oceanic islands
Source: Nat Commun. 2018 Jul 31;9:2820. doi: 10.1038/s41467-018-05280-8 (PMC6068123; doi:10.1038/s41467-018-05280-8)
Supplement: Supplementary file 1 — Supplementary Information [file 41467_2018_5280_MOESM1_ESM.pdf]

Supplementary Information

Predictable evolution towards larger brains in birds colonizing oceanic islands

Sayol *et al*.

Contents

Supplementary Figures 1-6

Supplementary Tables 1-3

Supplementary References

Supplementary Figures

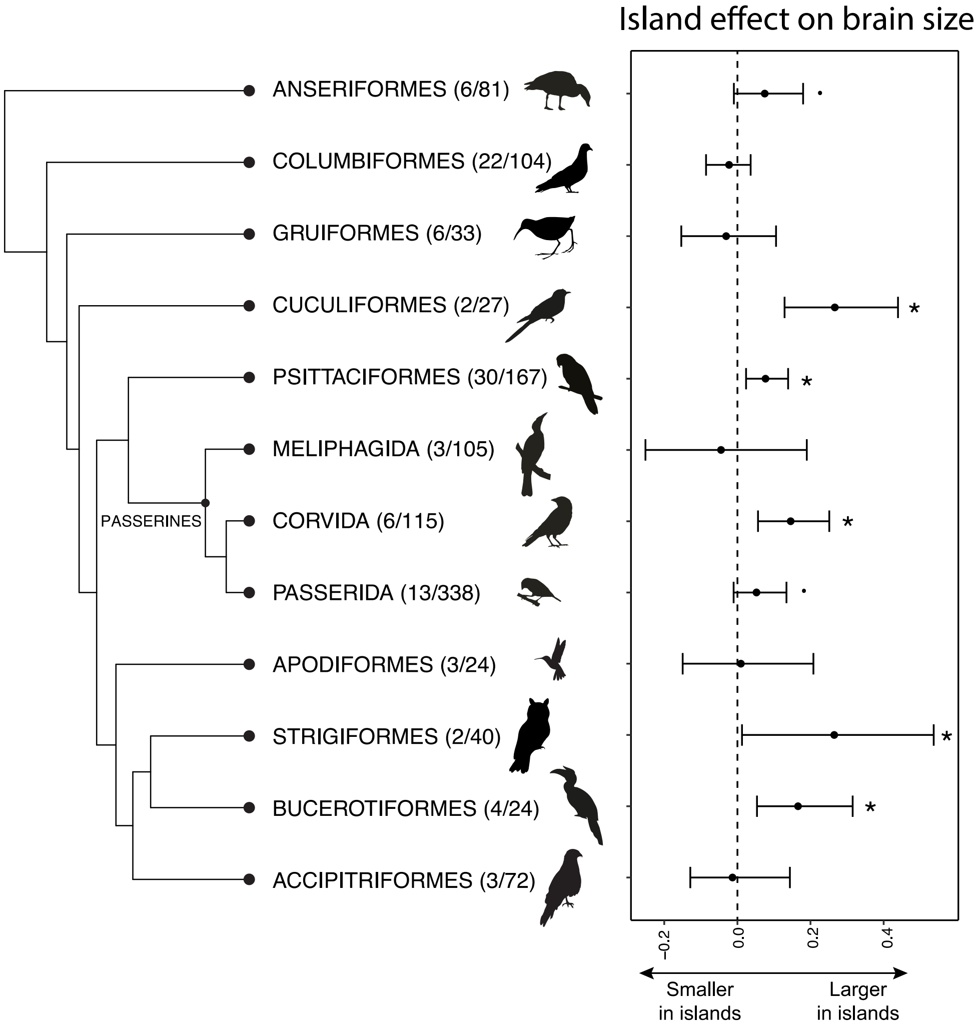

Supplementary Figure 1. Relative brain size differences between island and mainland species in different Avian clades. The posterior mode and 95% CI from a BPMM comparing the relative brain size of island and mainland species in each order are shown. The order Passeriformes was split into infraorders Meliphaga, Corvida and Passerida. (* pMCMC<0.05). The number of island (I) and mainland (M) species measured in each clade is given in parenthesis (I/M). The silhouettes were drawn by FS and are available at PhyloPic (http://phylopic.org).

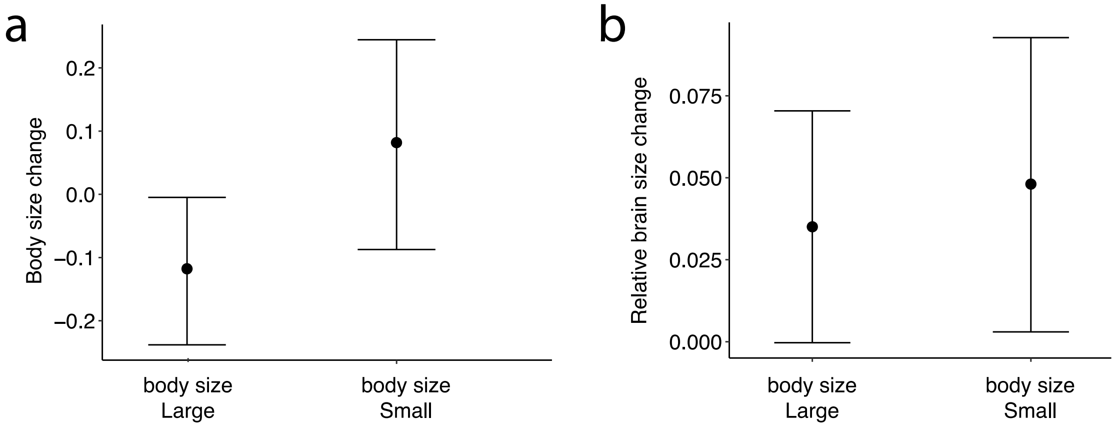

Supplementary Figure 2. Body size evolution does not explain why island species have relatively larger brains than mainland species. Although there exists an interaction between body size and the direction of body size changes (e.g. island rule) (**a**), both large and small birds tend to increase their relative brain size in islands (**b**) compared to the closest mainland sister species. The posterior mode and 95% CI from are shown.

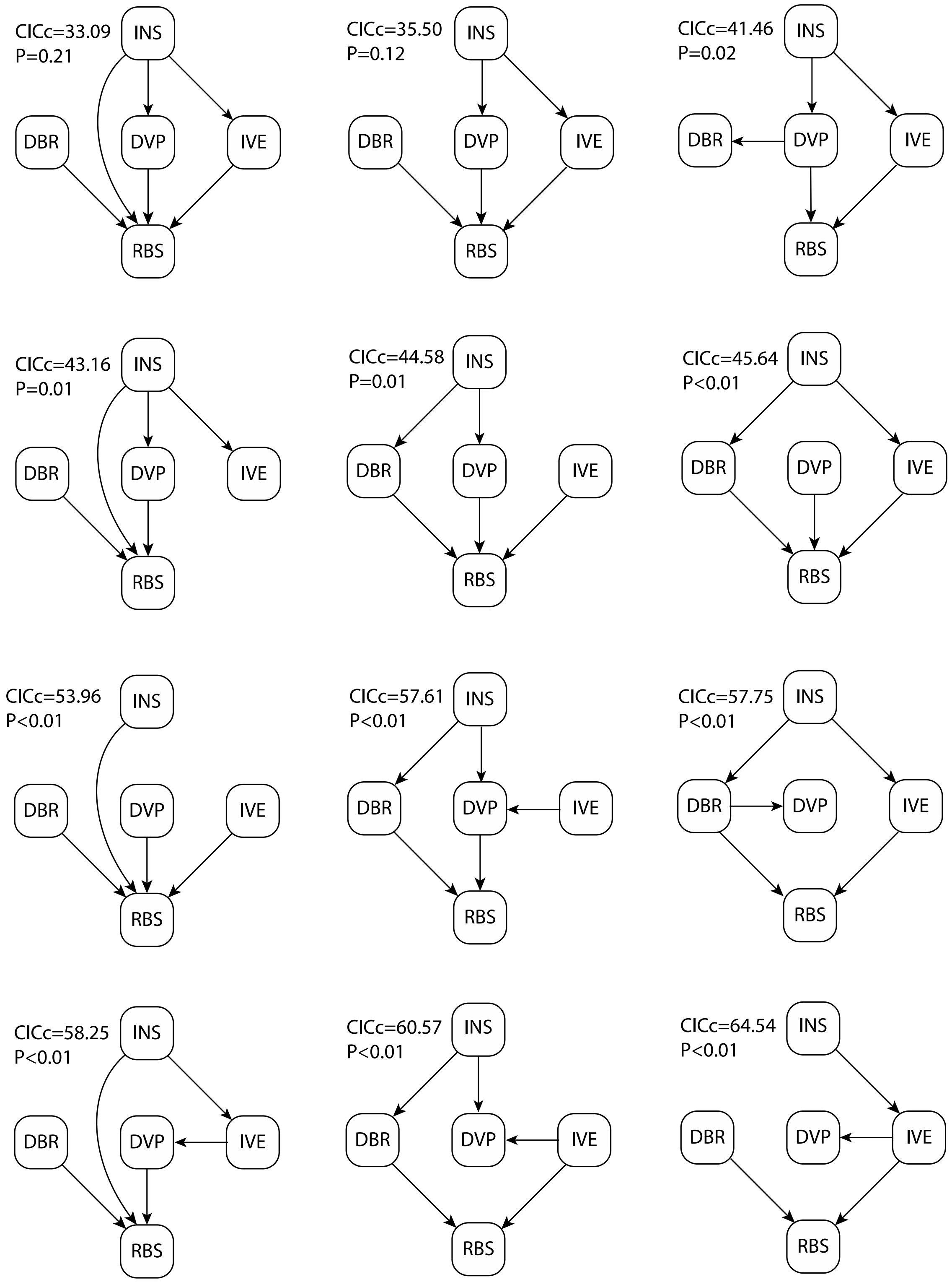

Supplementary Figure 3. Alternative models tested to explore the mechanisms underlying the relationship between relative brain size and island living. INS, Insularity; DBR, Diet breadth; DVP, Developmental period; IVE, Inter-annual variation in environmental productivity; RBS, Relative brain size.

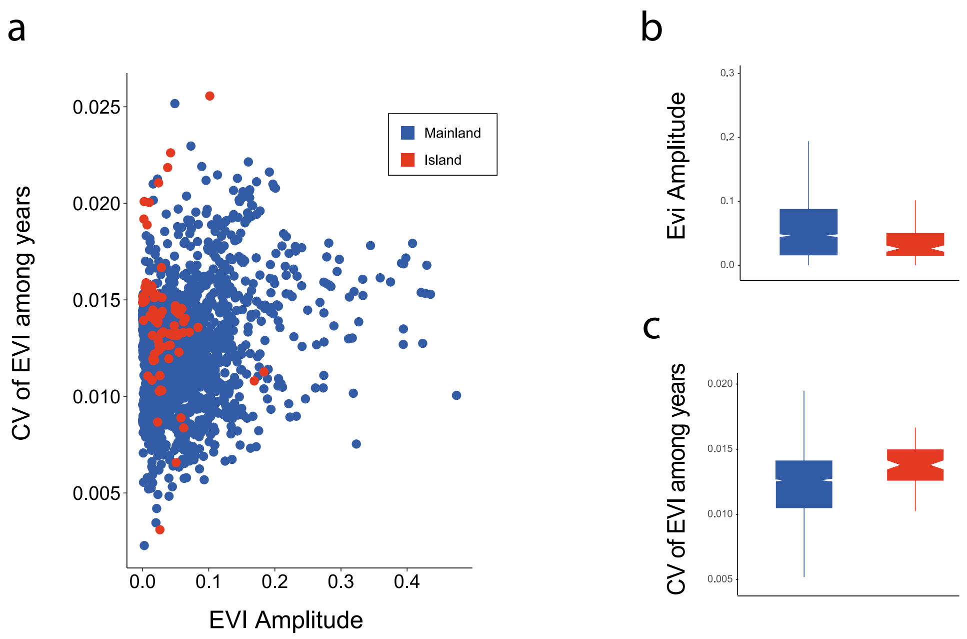

Supplementary Figure 4. Environment variation experienced by island species (red) compared with mainland species (blue). The scatterplot shows the relationship between the amplitude of variation in the enhanced vegetation index (EVI) within years and the coefficient of variation of EVI among years (a). The boxplots show the distribution (mean and percentiles [0.275, 0.25, 0.75 and 0.975]) of the same two variables (b, c) distinguishing island (red) and continental species (blue). Because potential homoscedasticity problems between the two environmental factors, we evaluated them separately in the path models.

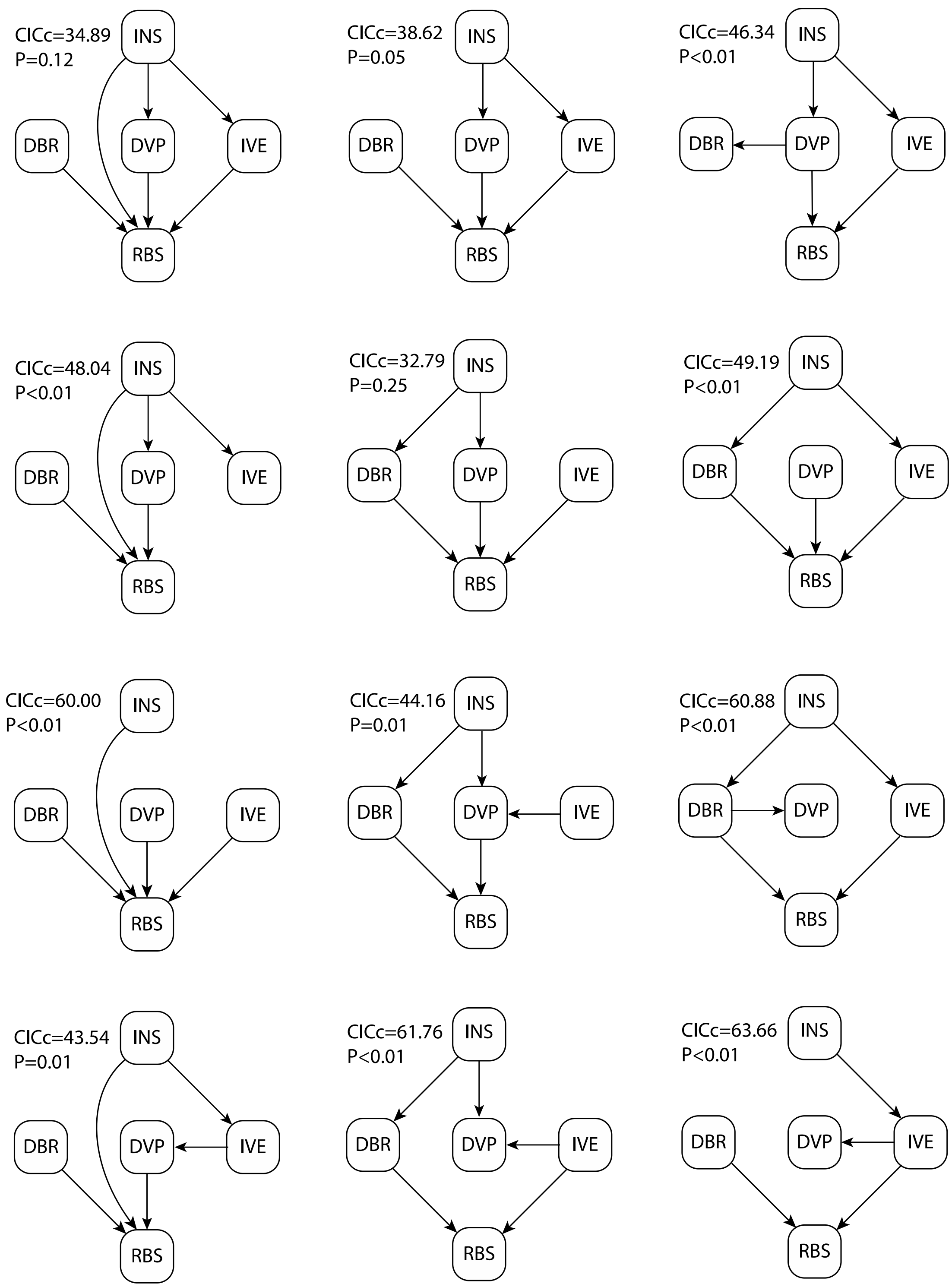

Supplementary Figure 5. Alternative models tested to explore the mechanisms underlying the relationship between relative brain size and island living. INS, Insularity; DBR, Diet breadth; DVP, Developmental period; SVE, Seasonal variation in environmental productivity; RBS, Relative brain size.

a

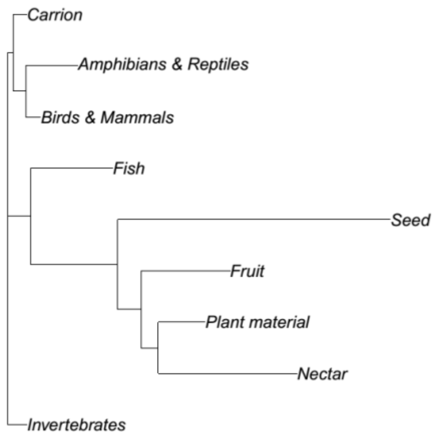

b

	P	G	F	L	W	O	Ref.
Carrion	16	0	0	5	75	4	(1)
Birds & Mammals	15	0	0	4.5	75.5	5	(1)
Amphibians & Reptiles	17.2	0	0	2.7	75.6	4.5	(2)
Fish	15	0	0	8	74.5	2.5	(3)
Invertebrates	17	0	3	5.5	70	4.5	(4-7)
Fruit	2	24	6.5	7	59	1.5	(8-9)
Seeds	9	28	44	3	8	8	(10-12)
Nectar	0	20.5	0	0	79.5	0	(13-14)
Plant material	4.5	26.5	8	3	55	3	(15-16)

Supplementary Figure 6. Resource similarity between food sources, used to calculate diet breadth index (**A**), based on nutritional content for each food item estimated from various sources (**B**) (P: Proteins; G: Carbohydrates- Glucides; F: Carbohydrates-Fiber; L: Lipids; W: Water content; O: Other).

Supplementary Tables

Supplementary Table 1. BPMMS of brain size (log-transformed) as a function of oceanic island living and body size including all the species of our study (model 1), only including species with at least 3 brain measurements (model 2), including interactions (model 3) and confounding factors (model 4). For migratory behaviour and developmental mode, residency and altriciality are respectively the reference levels.

Model	Response	Predictor	Estimate	95% CI	pMCMC
**Model 1** (N=1931)	Log(Brain)	Intercept	-2.511	[-2.725; -2.29]	<0.001
		Log (Body)	0.593	[0.583; 0.604]	<0.001
		Insularity	0.055	[0.028; 0.083]	<0.001
**Model 2** (N=1525)	Log(Brain)	Intercept	-2.534	[-2.754; -2.323]	<0.001
		Log (Body)	0.601	[0.588; 0.611]	<0.001
		Insularity	0.056	[0.022; 0.089]	0.002
**Model 3** (N=1931)	Log(Brain)	Intercept	-2.321	[-2.561; -2.089]	<0.001
		Log (Body)	0.593	[0.583; 0.603]	<0.001
		Insularity	0.046	[0.018; 0.074]	0.002
		Developmental mode (Precocial)	-0.257	[-0.362; -0.135]	<0.001
		Migratory behavior (Migrant)	-0.045	[-0.063; -0.028]	<0.001

Supplementary Table 2. BPMMS of relative brain size as a function of oceanic island living including each insular species and their closest continental taxa and an identifier of each comparison as a random factor in the model. For body size category, big size is the reference level (Nc=Number of comparisons).

Response	Predictor	Estimate	95% CI	pMCMC
**Model 1**				
Relative brain size	Intercept	0.190	[-0.128; 0.52]	0.128
(Nc=110)	Insularity	0.040	[0.011; 0.071]	0.005
**Model 2**				
Relative brain size	Intercept	0.197	[-0.124; 0.541]	0.122
(Nc=110)	Insularity	0.036	[-0.005; 0.074]	0.040
	Body category (Small)	-0.010	[-0.097; 0.076]	0.414
	Insularity : Body category (Small)	0.008	[-0.057; 0.078]	0.410
**Model 3**				
Relative brain size	Intercept	0.194	[-0.142; 0.53]	0.129
(Nc=110)	Insularity	0.039	[0.008; 0.071]	0.008
	Body category (Small)	-0.006	[-0.078; 0.068]	0.439

Supplementary Table 3. BPMMS of body size (log-transformed) as a function of oceanic island living and body size category including each insular species and their closest continental taxa and an identifier of each comparison as a random factor in the model. For body size category, big size is the reference level. (Nc=Number of comparisons).

**Response**	**Predictor**	**Estimate**	**95% CI**	**pMCMC**
Log(Body)	Intercept	6.713	[5.467; 8.083]	<0.001
(Nc=110)	Body category (Small)	-0.18	[-0.337; -0.005]	0.022
	Insularity	-1.185	[-1.526; -0.858]	<0.001
	Insularity : Small Body	0.275	[0.000; 0.586]	0.025

Supplementary References

1. Dierenfeld ES, Alcorn HL, Jacobsen KL, others (2002) Nutrient composition of whole vertebrate prey (excluding fish) fed in zoos (US Department of Agriculture, Agricultural Research Service, National Agricultural Library, Animal Welfare Information Center).
2. Özogul F, Yesim Ö, Olgunoglu AI, Boga EK (2008) Comparison of fatty acid, mineral and proximate composition of body and legs of edible frog (Rana esculenta). Int J Food Sci Nutr 59(7–8):558–565.
3. Bernard JB, Allen MA (1997) Feeding captive piscivorous animals: nutritional aspects of fish as food. Nutr Advis Gr Handbook Fact Sheet 5.
4. Oonincx D, Dierenfeld ES (2012) An investigation into the chemical composition of alternative invertebrate prey. Zoo Biol 31(1):40–54.
5. Fagbuaro O, Oso JA, Edward JB, Ogunleye RF (2006) Nutritional status of four species of giant land snails in Nigeria. J Zhejiang Univ Sci B 7(9):686–689.
6. Deblauwe I, Janssens GPJ (2008) New insights in insect prey choice by chimpanzees and gorillas in southeast Cameroon: the role of nutritional value. Am J Phys Anthropol 135(1):42–55.
7. Kinyuru JN, Kenji GM, Muhoho SN, Ayieko M (2010) Nutritional potential of longhorn grasshopper (Ruspolia differens) consumed in Siaya District, Kenya. J Agric Sci Technol 12(1):1–24.
8. Foster MS (1977) Ecological and Nutritional Effects of Food Scarcity on a Tropical Frugivorous Bird and its Fruit Source. Ecol Soc Am 58(1):73–85.
9. Herrera CM (1987) Vertebrate-dispersed plants of the Iberian Peninsula: a study of fruit characteristics. Ecol Monogr:305–331.
10. Kelrick MI, MacMahon JA, Parmenter RR, Sisson D V (1986) Native seed preferences of shrub-steppe rodents, birds and ants: the relationships of seed attributes and seed use. Oecologia 68(3):327–337.
11. Kerley GIH, Erasmus T (1991) What do mice select for in seeds? Oecologia 86(2):261–267.
12. Christian DP, Lederle PE (1984) Seed properties and water balance in desert granivores. Southwest Nat 29(2):181–188.

13. Baker HG (1975) Sugar concentrations in nectars from hummingbird flowers. Biotropica 7(1):37–41.
14. PYKE GH (1980) The foraging behaviour of Australian honeyeaters: a review and some comparisons with hummingbirds. Aust J Ecol 5(4):343–369.
15. McLandress MR, Raveling DG (1981) Changes in diet and body composition of Canada geese before spring migration. Auk 98(1):65–79.
16. Treichler R, Stow RW, Nelson AL (1946) Nutrient content of some winter foods of ruffed grouse. J Wildl Manage 10(1):12–17.
