# Supplementary material for: Predictable evolution towards larger brains in birds colonizing oceanic islands
Source: Nat Commun. 2018 Jul 31;9:2820. doi: 10.1038/s41467-018-05280-8 (PMC6068123; doi:10.1038/s41467-018-05280-8)
Supplement: Supplementary file 3 — Description of Additional Supplementary Files [file 41467_2018_5280_MOESM3_ESM.pdf]

Description of Additional Supplementary Files

File Name: **Supplementary Data 1**
Description: **Species data.** Dataset containing the list of species included in the study with the corresponding information on morphological, ecological and life-history traits.

File Name: **Supplementary Data 2**
Description: **Family data.** Dataset containing the list of families included in the study with the number of species living on island and continents, together with average brain size at the family level.
